# Exploring the Efficacy and Safety of Antidepressants in Bipolar Disorder: A Comprehensive Review

**DOI:** 10.1192/j.eurpsy.2025.1060

**Published:** 2025-08-26

**Authors:** M. Juhlin, D. Adedeji

**Affiliations:** 1 Solna Sundbyberg General Psychiatry department, Karolinska Institutet, Stockholm, Sweden; 2 General Psychiatry, Helgeland Hospital, Mosjøen, Norway; 3 Division of Clinical Geriatrics, Department of Neurobiology, Care Sciences, and Society, Karolinska Institutet, Stockholm, Sweden

## Abstract

**Introduction:**

Bipolar disorder (BD) is a complex mental health condition characterized by alternating periods of depression and mania, affecting millions worldwide. Despite its prevalence, the use of antidepressants, widely prescribed for unipolar depression, remains debated in the context of bipolar depression due to concerns about mood destabilization, mania induction, rapid cycling, and long-term efficacy and safety. This ambiguity underscores the critical need for a comprehensive analysis to guide clinical practice. This review aims to evaluate the efficacy, safety, and long-term outcomes of antidepressant use in bipolar disorder.

**Objectives:**

Assess the safety and efficacy of antidepressants in bipolar disorder. Optimize treatment options to help reduce the global burden of bipolar disorder and address a major gap in understanding regarding the role of antidepressants in treating bipolar disorder.

**Methods:**

A systematic review of 35 studies, including 18 randomized controlled trials (RCTs), 14 cohort studies, and 3 meta-analyses published between 2010 and 2023, was conducted. Studies were selected based on predefined inclusion and exclusion criteria, focusing on antidepressant efficacy, safety, and long-term effects in BD patients. Data extraction and synthesis followed rigorous methodological protocols. The extracted data were then analyzed to identify trends, themes, and contradictions in the literature.

**Results:**

The use of antidepressants in bipolar disorder should be highly individualized, balancing potential benefits against risks. Clinicians must exercise caution, particularly regarding the risk of mania induction. This review found that antidepressants, especially when combined with mood stabilizers, demonstrated moderate efficacy in treating bipolar depression. Outcomes varied significantly across studies; while some patients benefited from antidepressant use, others experienced increased risks, such as rapid cycling and mania induction. There is a lack of conclusive long-term safety data, highlighting the need for personalized treatment approaches to mitigate risks.

**Image:**

**Figure:**
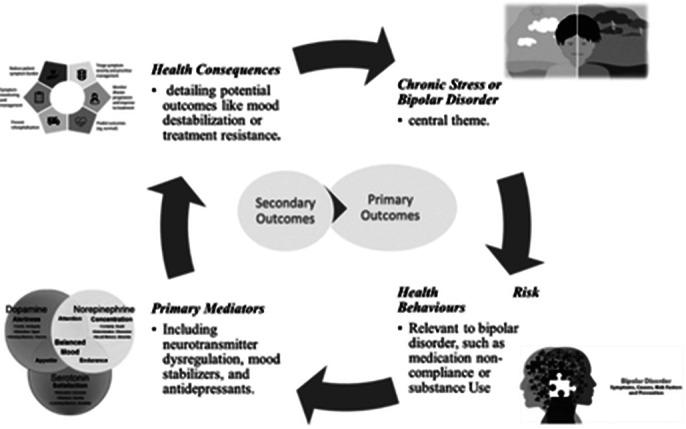

**Image 2:**

**Figure:**
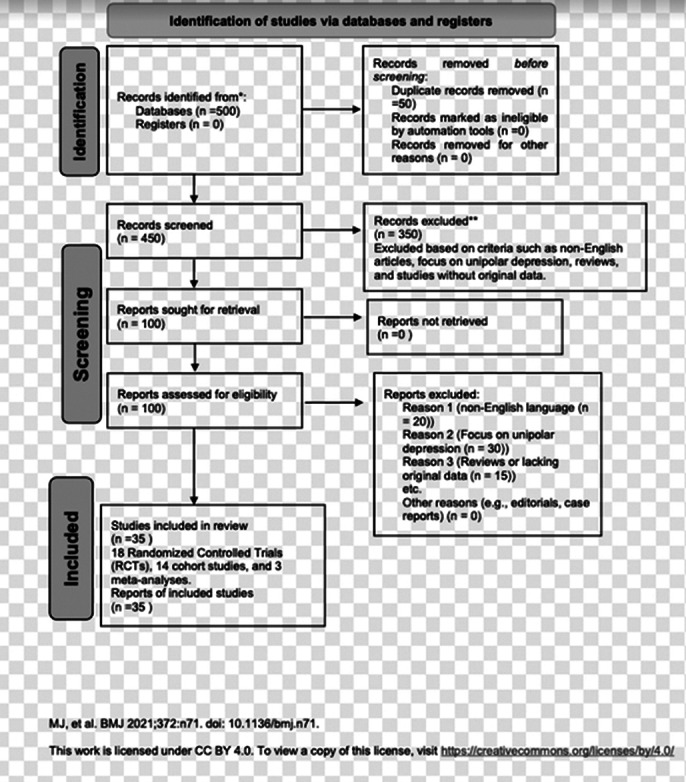

**Conclusions:**

The role of antidepressants in bipolar disorder treatment remains contentious due to variability in outcomes and safety concerns. A personalized treatment approach, incorporating mood stabilizers, is recommended. This literature review concluded it is essential to balance the benefits and risks. Therefore, a combined treatment regimen with mood stabilizers is recommended. Further research, particularly longitudinal studies, is necessary to establish more definitive, evidence-based guidelines for treating bipolar depression with antidepressants.

**Disclosure of Interest:**

None Declared

